# Improved Stability in LiX‐NbCl_5_ (X = Cl, Br) Glass‐Ceramic Electrolytes Through Anion Mixing for Solid‐State Batteries

**DOI:** 10.1002/smll.74224

**Published:** 2026-06-17

**Authors:** Jensheer Shamsudeen Seenath, Marvin Szabo, Philip Henkel, Rajib Sahu, Ramon Zimmermanns, Dhanush Yashwant Shanbhag, Christian Kübel, Stefanie Dehnen, Aleksandr Kondrakov, Torsten Brezesinski, Florian Strauss

**Affiliations:** ^1^ Battery and Electrochemistry Laboratory (BELLA) Institute of Nanotechnology Karlsruhe Institute of Technology (KIT) Karlsruhe Germany; ^2^ Institute of Nanotechnology Karlsruhe Institute of Technology (KIT) Karlsruhe Germany; ^3^ Institute For Applied Materials‐Energy Storage Systems Karlsruhe Institute of Technology (KIT) Karlsruhe Germany; ^4^ Helmholtz Institute Ulm (HIU) Ulm Germany; ^5^ Karlsruhe Nano Micro Facility (KNMFi) Karlsruhe Institute of Technology (KIT) Karlsruhe Germany; ^6^ Institute of Materials Science Technical University Darmstadt (TUDa) Darmstadt Germany; ^7^ BASF SE Ludwigshafen Germany

**Keywords:** amorphous solid, battery, electrochemistry, ionic conductivity, solid electrolyte

## Abstract

The realization of solid‐state batteries (SSBs) hinges upon the development of solid electrolytes (SEs) exhibiting superior functional properties. Halide SEs are promising candidates due to their high room‐temperature ionic conductivity and favorable (chemo)mechanical properties. However, their electrochemical stability and degradation processes under operating conditions remain largely unexplored. Herein, we present lithium niobium halide SEs, LiX‐NbCl_5_ (X = F^−^, Cl^−^, Br^−^, I^−^), with emphasis placed on LiNbCl_6_ and LiNbCl_5_Br. Structural analysis unveils the materials to be predominantly amorphous, interspersed with nanocrystalline domains, with both LiNbCl_6_ and LiNbCl_5_Br exhibiting ionic conductivities above 3.5 mS cm^−1^ at 25°C. Mechanical properties and pressure‐dependent ionic conductivities were also examined, revealing good densification behavior and low activation volumes. When used as catholyte in SSBs with layered oxide cathodes, the cells show high initial Coulomb efficiencies (>90%) and deliver specific discharge capacities of over 200 mAh g^−1^. Using differential electrochemical mass spectrometry, we demonstrate that chlorine evolves at the end of charge, which can be mitigated to some extent by introducing bromine, leading to enhanced cyclability. Overall, our study indicates that halide substitution has a positive effect on electrochemical stability without impairing ionic conductivity, and that gas evolution must be considered in halide‐based SEs.

## Graphical abstract

Chlorine gas evolves at the end of charge in solid‐state batteries with LiNbCl_5_X (X = Cl^−^, Br^−^) catholytes. This can be mitigated to some extent by introducing bromine and has a positive effect on electrochemical stability but does not impair ionic conductivity. Gas evolution must be considered in the future design of halide solid electrolytes.

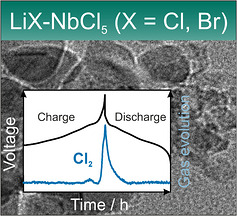

## 1 Introduction

The pursuit of safer and more energy‐dense storage solutions has positioned solid‐state batteries (SSBs) at the forefront of battery innovation [1, 2, 3]. As conventional lithium‐ion technologies approach their theoretical limits, SSBs offer a promising pathway toward overcoming existing constraints in energy density, safety, and cycle life. Central to this paradigm shift is the development of solid electrolytes (SEs) that combine high ionic conductivity, requisite mechanical compliance for scalable cell manufacturing, a wide operational voltage window, robustness against humidity, and economic viability [4, 5, 6].

In the landscape of inorganic SEs, broadly categorized into oxides [7, 8], sulfides [9, 10, 11], and halides [12, 13], the latter emerge as particularly promising candidates due to their favorable combination of properties. Specifically, chloride‐based SEs, such as Li_3_MCl_6_ (where M = In^3+^, Sc^3+^, Y^3+^, etc.), demonstrate excellent (electrochemical) oxidation stability, enabling direct integration with high‐voltage cathode active materials (CAMs) without the need for protective surface coating [14]. This compatibility stands in contrast to the interfacial challenges plaguing other SE families, i.e., thiophosphates. However, the ionic conductivities achieved in crystalline halides have generally trailed those of sulfide SEs, thus limiting their immediate use in applications demanding high power output or rapid charging capabilities [15, 16]. This conductivity bottleneck is often rooted in the close‐packed nature of their anion lattices, which present substantial energy barriers for lithium migration [17]. Consequently, a significant research thrust is directed toward rationally enhancing halide conductivity through the design of novel compositions and structural architectures, leveraging both computational modeling and advanced synthetic methodologies [18, 19, 20].

Recent investigations have illuminated the intriguing potential of amorphous or glass‐ceramic halides, exemplified by LiTaCl_6_ [21], although the precise mechanisms underpinning their high ionic conductivity remain largely elusive up until now [22]. Aside from such purely halide‐based SEs, a particularly promising development involves the synthesis of lithium oxyhalide materials, achieved through the incorporation of oxygen anions within halide structures [23, 24]. These materials display structural characteristics containing both crystalline and amorphous fractions and are able to achieve ionic conductivities in the range of 10^−3^ to 10^−2^ S cm^−1^ at 25°C, coupled with compelling battery performance [25]. Hence, there's great potential for tailoring the properties of this class of glass‐ceramics by synthesis conditions and compositional variations. Beyond the critical aspect of ionic conductivity, the successful realization of SSBs necessitates addressing other significant challenges. These include mitigating interfacial impedance between SE and electrode active material and ensuring robust solid‐solid contact, especially for high mass loading/capacity cathodes [26, 27, 28]. While crystalline (ceramic) SEs across oxide, sulfide, and halide families have exhibited promising bulk conductivities [29], their practical deployment in SSBs is often hindered by the intrinsic rigidity of materials, making it challenging to form dense cathode composites and maintain electrode integrity during cycling, where continuous volume changes induce (chemo)mechanical strain. In contrast, amorphous or glass‐ceramic SEs offer an inherent advantage in achieving intimate interfacial contact due to their flexible structure and facile lithium conduction pathways [29]. Amorphous sulfide‐ and oxysulfide‐based SEs offer higher conductivities [30] but often suffer from electrochemical instabilities, particularly at high potentials [31, 32]. Soft halides represent a class of SEs that possibly combine the advantages of electrochemical stability with mechanical softness. This unique combination addresses one of the most persistent challenges in SSB development, maintaining intimate contact at the electrode|electrolyte interface during cycling. The inherent deformability of these materials facilitates favorable ion percolation throughout the cathode, which is crucial for practical batteries.

Despite recent progress in halide‐based SEs, a fundamental understanding of their electrochemical stability and degradation pathways under realistic battery operation remains limited. In particular, the role of halide chemistry in determining interfacial side reactions and gas evolution during cycling has not been systematically investigated. Herein, we report on lithium niobium halide (LiX‐NbCl_5_ with X = Cl^−^, Br^−^)‐based glass‐ceramic SEs having high ionic conductivities at 25°C. Our investigations, including temperature‐ and pressure‐dependent ionic conductivity studies, reveal low activation energies and activation volume characteristics. More importantly, cells assembled using a bilayer separator design, strategically pairing different SE chemistries (e.g., sulfide facing anode and halide facing cathode) [33, 34], delivered high capacities and exhibited good longevity. *In situ* gas analysis via differential electrochemical mass spectrometry (DEMS) further indicated that Br^−^ substitution helps mitigate the evolution of detrimental gaseous side products, a finding that directly correlates with improved cycling performance. Overall, the insights presented here will help advance the understanding and development of high‐performance, halide‐based (soft) SEs for next‐generation battery applications.

## 2 Results and Discussion

Initially, we have investigated the mechanochemical reaction between NbCl_5_ and lithium halides, LiX (X = F^−^, Cl^−^, Br^−^, I^−^), forming materials with the nominal composition of LiX‐NbCl_5_ or LiNbCl_5_X. The resulting X‐ray diffraction (XRD) patterns are presented in Figure S1a, exhibiting diffuse background contributions among a few broad reflections, thereby indicating mostly amorphous structures. Preliminary measurements of ionic conductivities on cold‐pressed pellets at 25°C revealed values of 0.017 and 0.026 mS cm^−1^ for X = F^−^ and I^−^, respectively (Figure S2a). Moreover, we note that, for X = F^−^, achieving complete reaction seems challenging, as minor reflections associated with the LiF precursor were still detected after extensive milling. This suggests that the mechanochemically initiated reaction of NbCl_5_ with LiF is sluggish, which is likely related to the hardness of LiF and/or the electronegativity of fluorine [35], which forms strong ionic bonds with lithium, and therefore, is not susceptible to reacting with NbCl_5_. For X = I^−^, we observed an immediate reaction with NbCl_5_ already during hand grinding, resulting in a black powder, indicative of undesired redox reactions accompanied by the reduction of Nb^5+^ and formation of iodine. In contrast, for X = Cl^−^ and Br^−^, similar XRD patterns were found and, more importantly, the ionic conductivities at 25°C were well above 1 mS cm^−1^ (Figure S2b). Hence, in the following, we only focus on the samples with X = Cl^−^ and Br^−^.

To study the effect of thermal treatment on the structural and charge‐transport properties of LiNbCl_6_, the sample was annealed at 100°C under vacuum. The respective XRD pattern (Figure S1b) retained the features of the non‐heated sample but displayed a shift in peak positions, suggesting subtle changes to the crystalline environment. However, this kind of structural modification already led to a decrease in ionic conductivity by about 50% compared to the as‐prepared LiNbCl_6_, indicating that even minor changes in structure may negatively affect ion mobility. Annealing at 200°C resulted in the exsolution of a significant fraction of LiCl, causing a substantial drop in ionic conductivity, consistent with previously reported observations on amorphous, halide‐based SEs [23]. This phase separation is detrimental because of the loss of mobile Li^+^ ions and microstructural degradation due to the formation of insulating LiCl, disrupting the continuous conduction pathways within the amorphous network.

**FIGURE 1:**
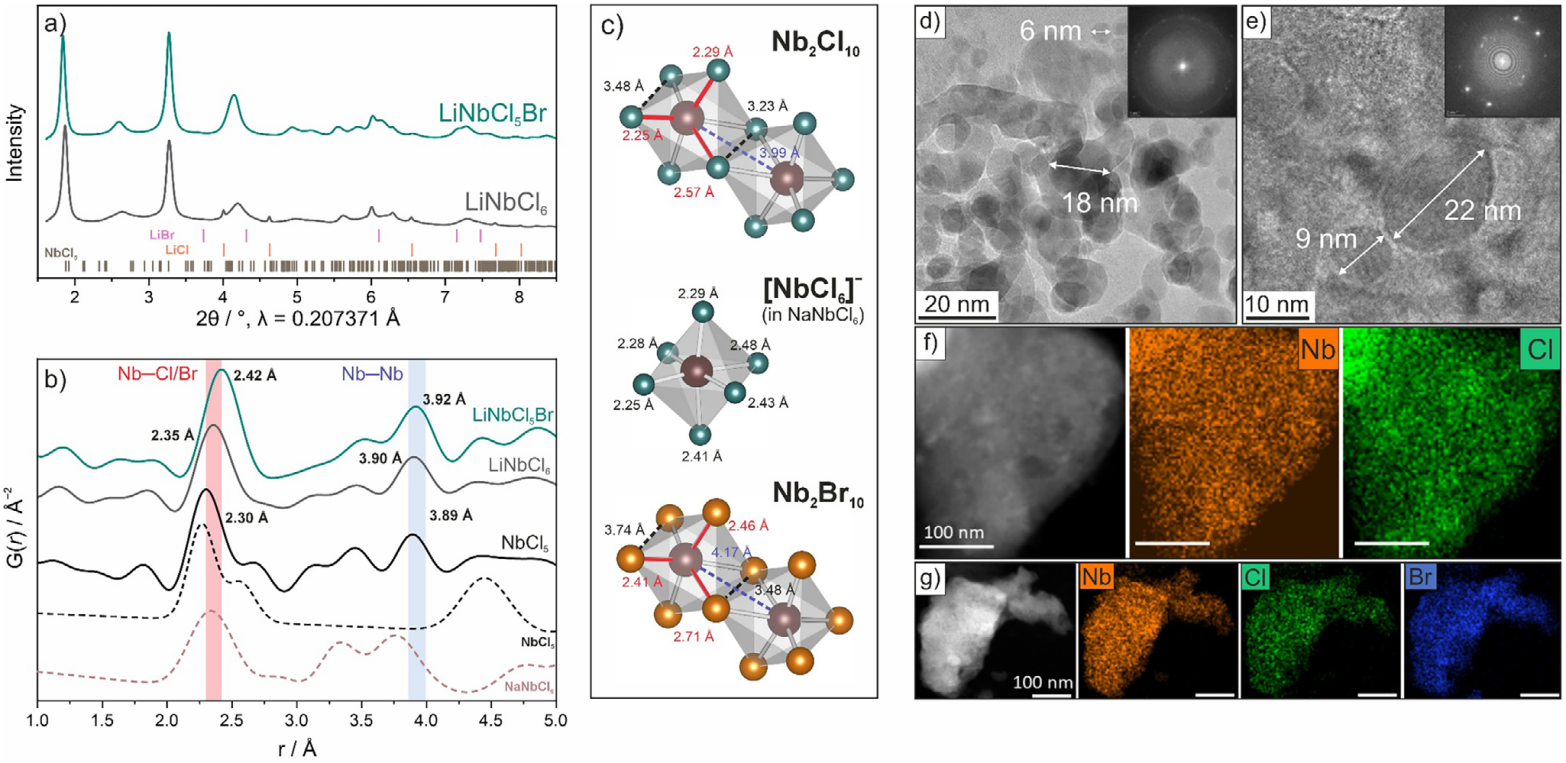
Structural characterization of the lithium niobium halide glass‐ceramic solid electrolytes. (a) SXRD patterns of LiNbCl_6_ and LiNbCl_5_Br. Vertical bars indicate the position of Bragg reflections for NbCl_5_, LiCl, and LiBr. (b) PDF analysis extending to 5 Å, illustrating the local atomic correlations in the NbCl_5_ precursor and the synthesized samples. Reference PDFs for NbCl_5_ and NaNbCl_6_ are given for comparison. (c) Schematic representations of the structural units around niobium in NbBr_5_, NbCl_5_, and [NbCl_6_]^−^ (present in NaNbCl_6_ for comparison) taken from the inorganic crystal structure database (ICSD). TEM images of (d) LiNbCl_6_ and (e) LiNbCl_5_Br with their corresponding FFTs shown as insets. STEM‐EDS elemental distribution maps of (f) Nb and Cl in LiNbCl_6_ and (g) Nb, Cl, and Br in LiNbCl_5_Br.

The structural characteristics of the as‐synthesized LiNbCl_5_X (X = Cl^−^, Br^−^) SEs were then investigated in some more detail using synchrotron XRD (SXRD) and pair distribution function (PDF) analysis of the total scattering data. As shown in Figure 1a, the SXRD patterns of the two compositions predominantly exhibit broad reflections, which decrease strongly in intensity with increasing scattering angle. SXRD peak deconvolution was employed to estimate the crystallinity of LiNbCl_6_ and LiNbCl_5_Br to be 21 and 32%, respectively, indicating that the major fraction is amorphous in nature. Aside from that, trace impurity peaks, likely originating from residual LiCl, were observed in the case of LiNbCl_6_. Moreover, the materials have a poor thermal stability, as illustrated exemplarily for LiNbCl_6_ in Figure S1b, where decomposition already occurred upon annealing at 200°C. This sensitivity underscores the importance of low‐temperature processing and storage conditions for glass‐ceramic (halide‐based) SEs. However, the mostly amorphous nature of the samples prevented us from thoroughly analyzing structural features. Moreover, SEM analysis (Figure S3) revealed that the SEs possess irregular morphologies and relatively broad particle size distributions, characteristic of mechanochemically synthesized glass‐ceramics.

To gain insights into the local atomic arrangement, the total scattering data were subjected to PDF analysis (shown for a real‐space distance up to 5 Å in Figure 1b). At first glance, all PDFs present a similar shape, indicating similar atomic arrangements. The prominent first peak located at 2.3 Å for NbCl_5_, 2.35 Å for LiNbCl_6_, and 2.42 Å for LiNbCl_5_Br can be attributed to the nearest‐neighbor Nb‒Cl/Br bond distances within the coordination environment of niobium. These bond distances are in good agreement with those for NbCl_5_/NbBr_5_ and NaNbCl_6_ taken from the inorganic crystal structure database (ICSD), see Figure 1c. Note that NbCl_5_ and NbBr_5_ consist of chlorine‐ or bromine‐bridged dimers. However, the better agreement of the simulated PDF for NaNbCl_6_ with the experimental data of both SEs indicates the presence of isolated [NbCl_6_]^−^ octahedral building units rather than bridged dimers. The observed shift toward larger distances upon reaction of NbCl_5_ with LiBr, in contrast to LiCl, suggests some expansion of the Nb‐centered polyhedra within the structure. The Nb‒Nb correlations, observed at about 3.9 Å, also exhibited a slight shift compared to the NbCl_5_ precursor, corroborating the alteration in intermediate‐range ordering and local bonding environment within the SE framework. In addition, for LiNbCl_5_Br, the Nb‒Nb distance is noticeably too short, suggesting closer interactions than those in NbCl_5_ and NbBr_5_. A summary of the key interatomic distances is given in Table S1. The PDF data spanning the complete range up to 13 Å (Figure S4) revealed well‐defined peaks in the short‐range region (1–5 Å), corresponding to preserved local coordination environments, with the rapid damping of intensities at higher *r*‐values (>5 Å) confirming the largely amorphous nature of both materials. The PDF data collected from the precursor materials is also shown in Figure S4, and virtually no evidence of remaining features related to the binary lithium halides was found for LiNbCl_5_X.

Transmission electron microscopy (TEM) imaging combined with fast Fourier transformation (FFT) analysis of the LiNbCl_6_ and LiNbCl_5_Br samples (Figure 1d,e) provided direct evidence of their biphasic nature. The latter is also evident from the selected‐area electron diffraction (SAED) patterns presented in Figure S5, showing diffuse scattering for the amorphous regions and sharper diffraction rings and spots for the nanocrystalline domains. Due to the poor scattering intensities, the calculation of lattice parameters was not possible. However, the layer spacing of the nanocrystalline domains was determined to be 2.12 and 3.2 Å for LiNbCl_6_ and LiNbCl_5_Br, respectively (Figure S5). The images indicate the size of the nanocrystalline domains to range from 3 to 25 nm. The lack of larger crystallites is consistent with the broad reflections and diffuse background observed in the XRD patterns. Energy‐dispersive X‐ray spectroscopy (EDS) mapping (Figure 1f,g) further indicated the presence of the accessible elements (Nb, Cl, and Br) in the analyzed regions, suggesting incorporation of all relevant species into the glass‐ceramics. Integrated spectra with the element‐specific characteristic peaks denoted are illustrated in Figure S6, and TEM images at different magnifications for both materials are provided in Figure S7.

**FIGURE 2:**
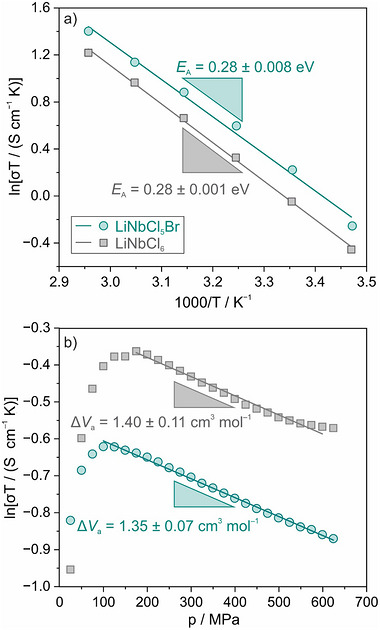
Lithium transport properties of LiNbCl_6_ and LiNbCl_5_Br. (a) Arrhenius plots with corresponding activation energies. (b) Pressure‐dependent ionic conductivity during decompression. The activation volumes of both solid electrolytes are given. Data is averaged from two independent measurements.

The ionic conductivities of the glass‐ceramic SEs were probed using temperature‐dependent electrochemical impedance spectroscopy (EIS) (Figure S8). Among the synthesized materials, LiNbCl_6_ and LiNbCl_5_Br showed promising ionic conductivities of (3.46 ± 0.22) mS cm^−1^ and (4.00 ± 0.20) mS cm^−1^ at 25°C, respectively. The corresponding Arrhenius plots are shown in Figure 2a, yielding activation energies (*E*_A_) of about 0.28 eV for both materials. These low *E*_A_ indicate facile lithium transport within the glass‐ceramics, and the determined ionic conductivities demonstrate slight benefits arising from halide substitution (anion disorder).

Further, the pre‐exponential factor (*σ*_0_) was extracted from Equation (1) to elucidate the observed differences in conductivity between the SEs, particularly given their similar *E*_A_.

(1) $$\sigma_{\mathrm{ion}}T=\sigma_{0\;}\exp\left(-\frac{E_{A}}{k_{B}T}\right)$$

In general, the ionic conductivity of solids (*σ*_ion_) is governed by a hopping process of ions, with the activation barrier *E*_A_ determining the mobility, as well as the Arrhenius pre‐exponential factor. The latter factor contains physical parameters that can be deconvoluted according to Equation (2).

(2) $$\sigma_{0}=\frac{\gamma n\nu a^{2}e^{2}}{k_{B}}\text{exp}\left(\frac{\Delta S_{m}}{k_{B}}\right)$$

where *γ* is the geometrical factor, *n* the charge‐carrier concentration, *ν* the attempt frequency, *a* the hopping distance, Δ*S*_m_ the migration entropy, *e* the elementary charge, and *k*_B_ represents the Boltzmann constant. The average *σ*_0_ values extracted were (5.78 ± 1.05) ∙ 10^4^ S cm^−1^ K for LiNbCl_6_ and (5.23 ± 2.15) ∙ 10^4^ S cm^−1^ K for LiNbCl_5_Br. While the significance of activation energy becomes prominent at elevated temperatures, the pre‐exponential factor dominates the conductivity behavior at low to moderate temperatures. A slightly higher average *σ*_0_ value for LiNbCl_6_, despite (assumed) similar charge‐carrier concentrations, might suggest enhanced structural disorder in the case of LiNbCl_5_Br [36].

Apart from *E*_A_, the activation volume (Δ*V*_a_) can be calculated from the pressure dependence of the ionic conductivity by Equation (3) [37, 38]. Physically, Δ*V*_a_ can be viewed as the volume change when an ion jumps from the ground state (equilibrium position) to the transition state (saddle point). A more typical definition is the difference between the volume an ion needs at the transition site during migration (*V*_m_) and the already available free crystallographic volume (*V*_f_) in the structure. Therefore, Δ*V*_a_ indicates whether an ion migration process needs a local expansion (positive Δ*V*_a_) or contraction (negative Δ*V*_a_) of the lattice [39, 40, 41, 42]. In principle, the activation volume can be obtained from the following pressure‐dependent conductivity relationship:

(3) $$\Delta V_{a}\approx -k_{B}T\;{\left[\frac{\partial \ln\left(\sigma T\right)}{\partial p}\right]}_{T}$$

Here, *σ* is the ionic conductivity, *p* the applied pressure, *k*_B_ the Boltzmann constant, and *T* represents the temperature.

To further probe the mechanism of ion transport, pressure‐dependent ionic conductivities were determined for the LiNbCl_6_ and LiNbCl_5_Br samples. Usually, microscopic effects are prevailing at relatively lower pressures (i.e., powder compression, densification, and improved particle‐to‐particle contact), whereas atomistic effects take place at higher pressures (i.e., molar volume compression). Figure 2b shows the natural logarithm of ionic conductivity versus the applied pressure during decompression. During pressure release from 650 MPa, a linear increase in ionic conductivity occurred until about 100 and 200 MPa for LiNbCl_5_Br and LiNbCl_6_, respectively. Afterwards, a strong decrease in ionic conductivity with decreasing pressure was observed. From the linear response, the activation volumes were calculated. For LiNbCl_6_ and LiNbCl_5_Br, Δ*V*_a_ was determined to be (1.40 ± 0.11) cm^3^ mol^−1^ and (1.35 ± 0.07) cm^3^ mol^−1^, respectively. The ionic conductivity data for compression and decompression are presented in Figure S9. The observed behavior reflects both intrinsic material properties and extrinsic factors related to the microstructure. During initial compression, the ionic conductivity increased and leveled off at about 400 MPa for LiNbCl_6_ and 350 MPa for LiNbCl_5_Br, likely an effect of sample consolidation [43, 44]. Upon decompression from the fully compressed state (650 MPa), both materials showed a maximum conductivity around 100 MPa. Moreover, one can note substantial hysteresis between compression and decompression. In general, as mentioned previously, it can be assumed that microscopic effects dominate at low pressures, while atomistic effects come into play at high pressures. However, both can be differentiated, as microscopic effects are usually not reversible in nature, whereas atomistic effects are reversible, meaning that if there is hysteresis in conductivity during pressure increase and release, this strongly hints at a microstructure‐dominated regime [44, 45]. By contrast, if the measured conductivities superimpose, one can refer this regime to atomistic effects. From the pressure‐dependent conductivity data in Figure S9, it is apparent that there was no hysteresis at pressures beyond 350 MPa, while below the hysteresis became more pronounced with decreasing pressure. This suggests that atomistic and microscopic effects dominate at pressures above and below 350 MPa, respectively. Regardless, low Δ*V*_a_ values (<< 5 cm^3^ mol^−1^) strengthen our hypothesis that atomistic effects are responsible for the observed behavior. In line with our study, activation volumes have been measured for a variety of ion‐conducting glasses, in which the pressure was found to reduce the number of free (mobile) ions [46, 47]. The respective values fall in the same range as those previously reported for crystalline SEs. However, the pressure required to achieve a decrease in ionic conductivity was much lower (in the MPa range). Note that, for example, in the case of thiophosphate SEs, GPa are needed to detect a similar behavior [42]. This emphasizes the mechanically soft nature of halide‐based, glass‐ceramic SEs, which is potentially beneficial to accommodating volume variations of the CAM upon battery operation. The slightly lower Δ*V*_a_ observed for LiNbCl_5_Br might suggest a lower local volume change during ion migration, which agrees with its slightly higher ionic conductivity and potentially indicates the presence of less constrained diffusion pathways. Nevertheless, the effects that chemistry and microstructural changes have on the activation volume cannot be readily deconvoluted due to the rather complex phase composition of the SEs. Assuming that in the case of LiNbCl_5_X (X = Cl^−^ or Br^−^) microstructural differences do not affect the pressure versus conductivity behavior at high pressures, variations in activation volume are likely correlated to chemical composition, in particular to the substitution of Cl^−^ with larger Br^−^. Still, structural differences (phase composition) among the samples cannot be ruled out.

From the pressure‐dependent ionic conductivity measurements, the change in relative density during compression/decompression can be deduced, as illustrated in Figure S10. Notably, LiNbCl_6_ achieved much higher relative densities than LiNbCl_5_Br at low pressures, indicating higher deformability, with full densification around 500 MPa and a difference by 76% between the initial and fully decompressed states. By contrast, LiNbCl_5_Br revealed full densification toward the maximum applied pressure, with a difference in relative density by 100% before and after compression.

Cross‐sectional scanning electron microscopy (SEM) images of the SE pellets, taken after the pressure‐dependent ionic conductivity measurements, are presented in Figure S11. They show that LiNbCl_6_ exhibits significantly better densification, with minimal voids between individual particles, while LiNbCl_5_Br shows more pronounced porosity and interparticle gaps under identical pressing conditions. This enhanced densification behavior suggests a higher degree of plasticity or rearrangement within the glass‐ceramic structure under pressure, potentially contributing to improved interfacial contact in SSBs. It should be noted that no changes were noticed in the XRD patterns collected before and after compression.

The electronic partial conductivities were investigated via direct current (DC) polarization measurements by applying voltages from 0.3 to 0.5 V (Figure S12) and determined to be 9.9 ∙ 10^−7^ mS cm^−1^ and 6.4 ∙ 10^−5^ mS cm^−1^ for LiNbCl_6_ and LiNbCl_5_Br, respectively. These values are several orders of magnitude higher compared to sulfide and especially oxide SEs but seem to follow the trend that halides, regardless of being amorphous or crystalline, possess elevated electronic conductivities [48, 49, 50, 51]. The partially increased electronic conductivity, along with a high ionic conductivity, makes these materials attractive as catholytes, where mixed ionic‐electronic conductivity (MIEC) is desirable to enhance (electronic) percolation throughout the cathode composite.

**FIGURE 3:**
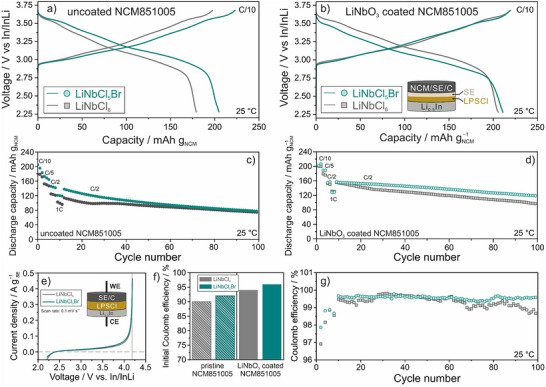
Electrochemical performance of LiNbCl_6_ and LiNbCl_5_Br as catholytes in SSB cells. Initial voltage profiles at C/10 rate for (a) uncoated NCM851005 and (b) LiNbO_3_‐coated NCM851005 cathodes using either LiNbCl_6_ or LiNbCl_5_Br. The inset in (b) shows a schematic of the cell design (NCM/SE/C: LiNi_0.85_Co_0.1_Mn_0.05_O_2_/solid electrolyte/Super C65 carbon). The SE in the separator layer facing the cathode was either LiNbCl_6_ or LiNbCl_5_Br, like the one in the cathode composite. (c, d) Rate capability (specific discharge capacities at C/10, C/5, C/2, and 1C) and long‐term cycling stability at C/2 for cells with (c) uncoated NCM851005 and (d) LiNbO_3_‐coated NCM851005 cathodes. (d) LSV curves for both SEs (WE: working electrode, CR: counter electrode). (f) Initial Coulomb efficiencies for the cells shown in (a) and (b). (g) Coulomb efficiencies corresponding to the cycling shown in (d). All data is averaged from two independent cells.

Initial charge‐discharge profiles of SSB cells with uncoated and LiNbO_3_‐coated LiNi_0.85_Co_0.1_Mn_0.05_O_2_ (NCM851005) as CAM and the respective glass‐ceramic SEs are illustrated in Figure 3a,b [10, 52, 53, 54]. Using the uncoated NCM851005, specific discharge capacities of *q*_dis_ ≈ 205 and 179 mAh g_NCM_^−1^ were achieved in the first cycle at C/10 and 25°C for LiNbCl_5_Br and LiNbCl_6_, respectively. Using the LiNbO_3_‐coated NCM851005, specific charge capacities of *q*_ch_ ≈ 219 mAh g_NCM_^−1^ were achieved with both SEs in the initial cycle. Upon discharge, the cells were found to deliver specific discharge capacities of *q*_dis_ ≈ 206 mAh g_NCM_^−1^ (LiNbCl_6_) and 211 mAh g_NCM_^−1^ (LiNbCl_5_Br). Overall, relatively high Coulomb efficiencies (CEs) of 90 and 92% were achieved with LiNbCl_6_ and LiNbCl_5_Br, together with the uncoated NCM851005 (Figure 3f). If the coated NCM851005 is applied, a further increase in first‐cycle CE is observed, leading to high values of 94 and 96% for LiNbCl_6_ and LiNbCl_5_Br, respectively, demonstrating that the protective surface coating on the CAM secondary particles helps reduce parasitic side reactions. Note that the mean discharge voltage of the LiNbCl_5_Br‐containing cells with the coated NCM851005 was slightly lower as compared to LiNbCl_6_, which might indicate the formation of a more resistive cathode‐electrolyte interphase (considering that the bulk ionic conductivity of both SEs is similar). The rate performance and long‐term cycling stability at C/2 of the uncoated and coated NCM851005 with both SEs are compared in Figure 3c,d. In cells using the uncoated NCM851005, both SE types exhibited an accelerated decline in specific discharge capacity during the long‐term cycling tests. In contrast, for the coated NCM851005, both SEs were found to enable high specific discharge capacities exceeding 200 mAh g_NCM_^−1^ at C/10. After rate capability testing, in which the different cells showed similar results, specific discharge capacities of about 160 mAh g_NCM_^−1^ were obtained at C/2. However, the LiNbCl_5_Br‐based cells demonstrated superior long‐term stability, retaining 76% of their initial capacity after 100 cycles, thus outperforming the LiNbCl_6_‐based cells, which only retained 62% under the same conditions.

The oxidative electrochemical stability of the SEs was probed using linear sweep voltammetry (LSV). As shown in Figure 3e, the onset of distinct anodic current flow was beyond 4 V vs. In/InLi (∼4.6 V vs. Li^+^/Li). While softer mechanical properties of LiNbCl_6_ explain the slightly better densification behavior, this does not directly translate to improved electrochemical stability, highlighting the importance of interfacial stability in determining the long‐term performance. The better electrochemical stability of the Br‐substituted SE is also evident from the evolution of CEs over cycling. For the LiNbO_3_‐coated NCM851005 cells, they stabilized near 99.5% after the initial cycles (Figure 3g). However, there was a clear decrease toward 99% for LiNbCl_6_, starting from the 60^th^ cycle.

A comparative summary of the electrochemical performance relative to state‐of‐the‐art SSBs using various halide‐based SEs and CAMs is presented in Table S2. Although halide SEs are typically considered to be anodically stable, recent research data indicates possible redox activity of high‐valent transition‐metal species, i.e., Nb^5+^ or Ta^5+^, which may negatively or positively impact battery performance depending on the reversibility [48, 55].

In summary, when uncoated NCM851005 is used as CAM, the LiNbCl_5_Br‐based cells are still capable of delivering a high initial specific discharge capacity, albeit with inferior rate performance and accelerated capacity fading compared to the LiNbO_3_‐coated counterpart. In general, the capacity retention is poor in this case (Figure 3c), highlighting the crucial role of the coating in stabilizing the cathode|electrolyte interface, even for lithium niobium halide SEs.

**FIGURE 4:**
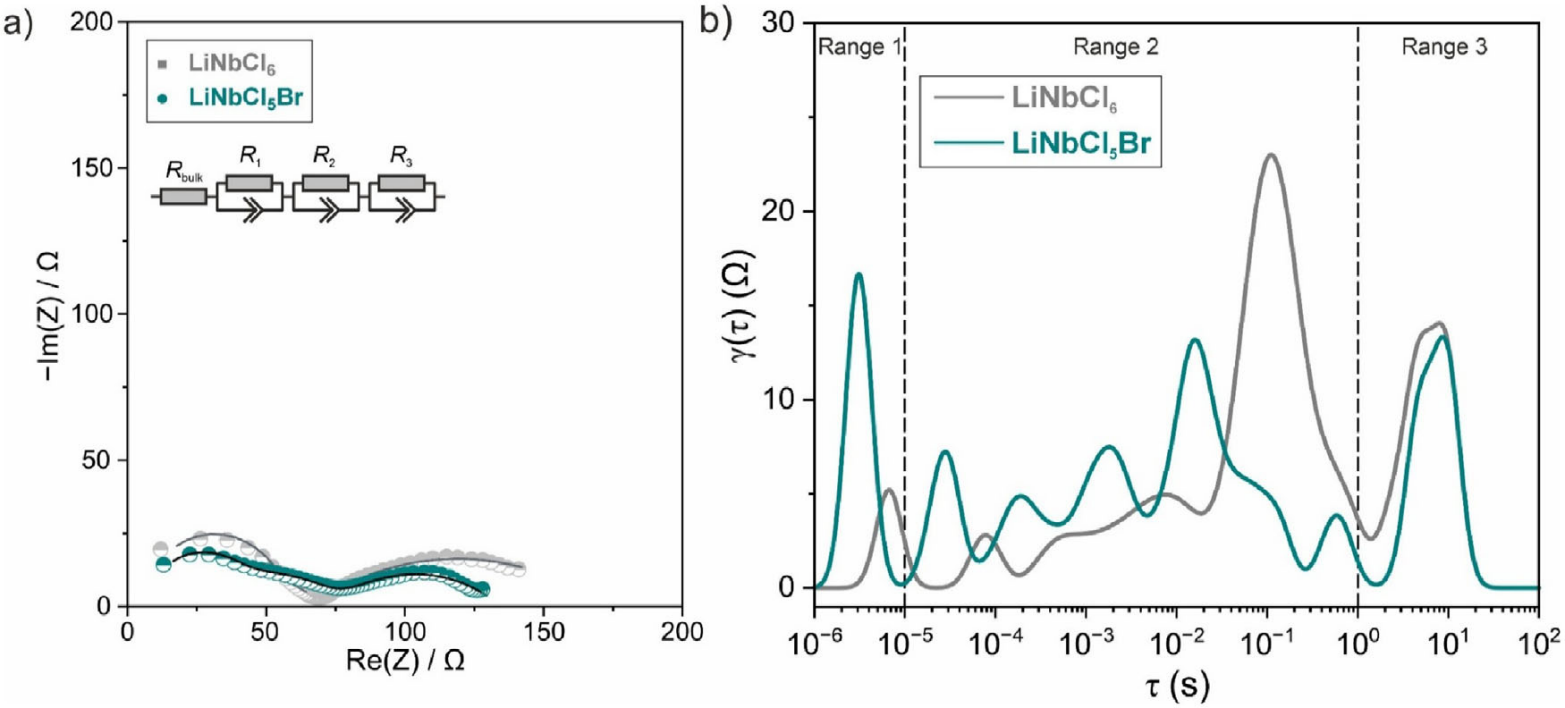
(a) Nyquist plots of the electrochemical impedance, calculated data using the equivalent circuit shown in the inset, and (b) corresponding DRT patterns of SSB cells with LiNbCl_6_ and LiNbCl_5_Br after the first charge. Range 1, with 10^−6^ to 10^−5^ s, refers to particle‐particle contact within the electrode or the contact resistance between electrode and current collector. Range 2, with 10^−5^ to 10^0^ s, can be correlated to a wide range of processes, including interphase formation and charge transfer over the as‐formed interfaces. However, due to time response overlap, it is virtually impossible to deconvolute every single process. Therefore, the calculated *τ* within this range has been assigned to the contribution of the interface without further distinguishing between multiple processes. Range 3, with > 10^0^ s, can be attributed to diffusion processes [4, 5].

*Ex situ* EIS measurements were also conducted on the LiNbO_3_‐coated NCM851005 cells to examine the impedance evolution and to reliably probe interfacial processes occurring between the CAM and the halide‐based SEs. Nyquist plots of the electrochemical impedance after the initial charge and results from distribution of relaxation times (DRT) analysis are presented in Figure 4a,b. DRT patterns typically reveal peaks associated with characteristic kinetic processes [56]: particle‐particle contact resistance within the cathode (*τ* ≈ 10^−6^ to 10^−5^ s), interphase resistance (*τ* ≈ 10^−5^ to 10^0^ s), charge‐transfer resistance (*τ* ≈ 10^−2^ to 10^0^ s), and bulk diffusion (*τ* ≈ 10^0^ to 10^2^ s). The impedance data was modeled using the equivalent circuit shown in the inset of Figure 4a. *R*_bulk_ represents the SE resistance and *R*_1_, *R*_2_, and *R*_3_ are interfacial impedances. The latter, however, are difficult to assign to individual anode and/or cathode contributions. Nevertheless, cells using LiNbCl_6_ exhibited a larger overall interfacial resistance of 166 Ω compared to those with LiNbCl_5_Br (129 Ω). The impedance buildup can likely be attributed to the formation of solid decomposition products at the cathode|electrolyte and/or carbon|electrolyte interface. Consequently, the better capacity retention achieved with LiNbCl_5_Br points toward a more robust decomposition interphase.

**FIGURE 5:**
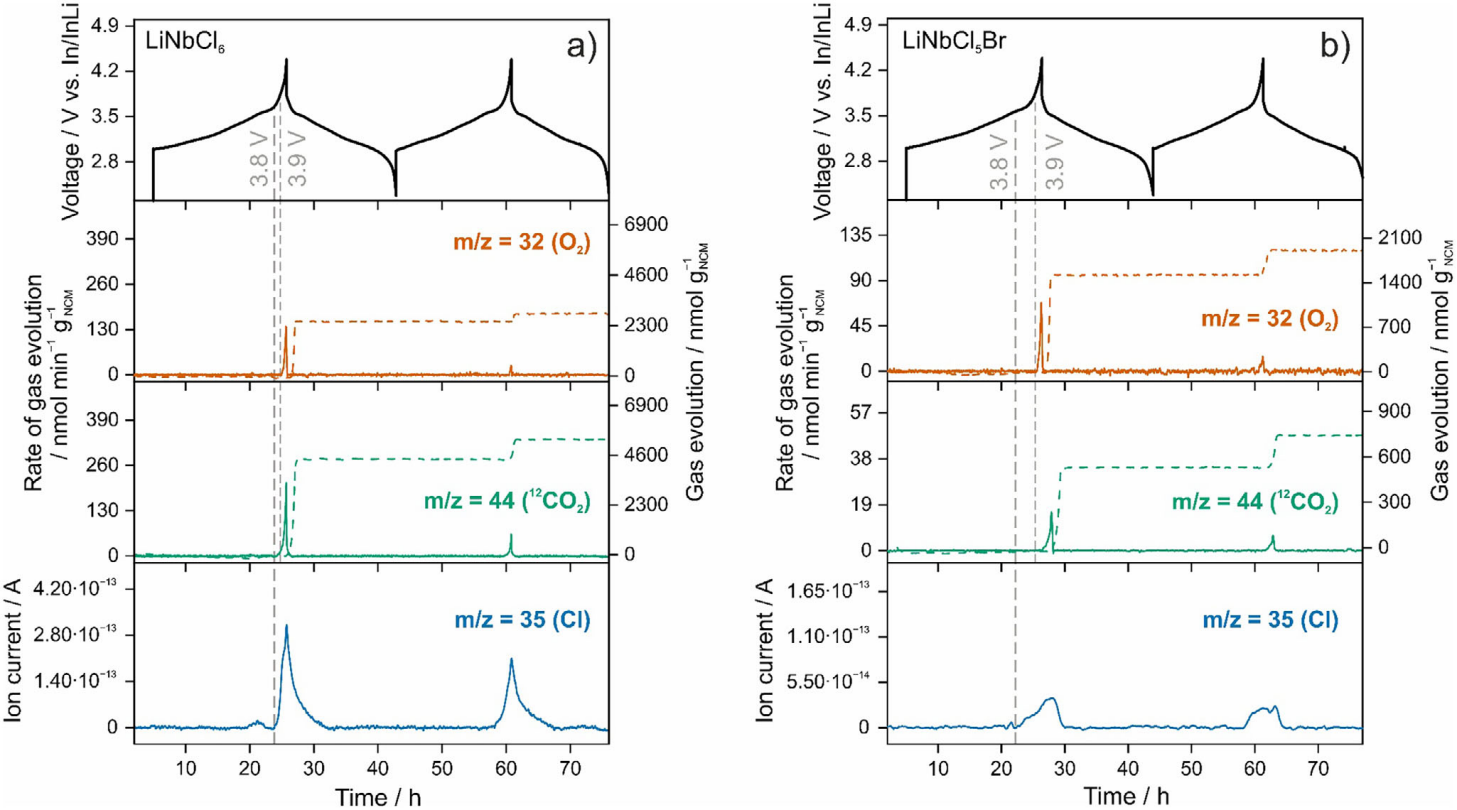
Voltage‐time profiles at C/20 of SSB cells using (a) LiNbCl_6_ and (b) LiNbCl_5_Br as catholytes and corresponding ^12^CO_2_ and O_2_ evolution rates as well as cumulative amounts of released gas and ion current for *m*/*z* = 35. The onset potentials for gas evolution are denoted by vertical dotted lines.

To examine the evolution of gaseous side products as one of the reasons for degradation and capacity decay during cycling [57, 58], DEMS measurements were conducted on the LiNbCl_6_‐ and LiNbCl_5_Br‐based cells. They were cycled at 25°C and at C/20 from 2.3 to 4.4 V vs. In/InLi (∼5.0 V vs. Li^+^/Li) and exhibited similar initial charge/discharge characteristics (Figure S13). Those containing LiNbCl_6_ delivered specific charge and discharge capacities of *q*_ch_ ≈ 197 mAh g_NCM_^−1^ and *q*_dis_ ≈ 162 mAh g_NCM_^−1^ in the first cycle, compared to 203 and 167 mAh g_NCM_^−1^ for LiNbCl_5_Br. The latter corresponds to a state‐of‐charge (SOC) of about 72% for LiNbCl_6_ and 74% for LiNbCl_5_Br (neglecting side reactions). Voltage‐time curves for the first two cycles are shown in Figure 5a,b, along with the DEMS signals for *m*/*z* = 32, 44, and 35, referring to O_2_, ^12^CO_2,_ and Cl, respectively. Of note, independent of the SE used, the onset of O_2_ and CO_2_ evolution occurred at about 3.9 V vs. In/InLi (∼4.5 V vs. Li^+^/Li) in the first and second cycles.

The O_2_ release is related both to the loss of oxygen from the CAM and to the electrochemical decomposition of carbonate residuals [59, 60]. Although it has been reported that an SOC above 80% is required for NCM‐type CAMs to release oxygen [61], in pelletized SSB cathode composites, inhomogeneities in SOC may be present during cycling. Therefore, the occurrence of CAM fractions possessing different SOC is feasible, ultimately leading to lattice O_2_ loss [62, 63, 64]. The electrochemical (oxidative) decomposition of Li_2_CO_3_ species present on the surface (and/or in the protective coating) accounts for CO_2_ and additional O_2_ release according to Equation (4) [58, 65]

(4) $$2Li_{2}CO_{3}\to 4Li^{+}+4e^{-}+\;2CO_{2}+O_{2}$$

Interestingly, we also detected signals for *m*/*z* = 35 for both SEs related to Cl species, indicating that upon the oxidative decomposition, gaseous chlorine is released with an onset potential of about 3.8 V vs. In/In Li (∼4.4 V vs. Li^+^/Li). To the best of our knowledge, this presents the first experimental evidence of chlorine being a product of (electro)chemical degradation of halide‐based SEs. As can be seen, distinct chlorine evolution was present at the end of the first as well as the second charge, however, being reduced in intensity for the latter. Specifically, the measured ion current for the Cl species was lower by about an order of magnitude for LiNbCl_5_Br. Although one can expect reduced chlorine release upon substitution, the ion currents indicate a stabilizing effect of bromine on the electrochemical stability. Note that the applied ionization current in the mass spectrometer reached high values so that all chlorine species, i.e., diatomic chlorine gas (Cl_2_), that evolve during cycling dissociate into Cl^+^ species, which are detected as *m*/*z* = 35. This observation provides clear experimental evidence that halide‐based SEs are not electrochemically inert under realistic cycling conditions. Importantly, partial substitution of Cl^−^ with Br^−^ leads to a measurable suppression of Cl_2_ evolution, suggesting that anion chemistry plays an important role in modulating degradation pathways, either involving solid or gaseous decomposition products.

Quantitative analysis was only possible for O_2_ and CO_2_. Pronounced O_2_ evolution of ∼2900 nmol g_NCM_^−1^​ was detected for LiNbCl_6_, compared to ∼1900 nmol g_NCM_^−1^ for LiNbCl_5_Br. A similar trend was observed for the CO_2_ evolution, with LiNbCl_6_ reaching ∼5500 nmol g_NCM_^−1^, whereas the LiNbCl_5_Br‐based cells released only ∼750 nmol g_NCM_^−1^. Collectively, these results demonstrate that SSBs with LiNbCl_5_Br release less gaseous decomposition products (i.e., CO_2_ and Cl) during cycling as compared to LiNbCl_6_. The amounts of evolved O_2_ were virtually similar, implying that the O_2_ released from the NCM851005 lattice does not undergo follow‐up solid‐gas reactions with the SEs. However, this might not be true for CO_2_, as the cumulative amount after two cycles was more than 7 times higher for the LiNbCl_6_‐based cells. Overall, the lower amounts of gases evolved in cells using LiNbCl_5_Br as catholyte is consistent with its better cycling stability, highlighting the beneficial role of bromine in improving (electro)chemical stability.

The formation of gaseous species can also negatively affect interfacial contact and contribute to the growth of resistive interphases, as confirmed by the increase in interfacial impedance observed using EIS (Figure S14). These findings indicate that interfacial degradation is the dominant factor governing capacity fading.

## 3 Conclusion

In summary, we attempted to synthesize a series of lithium niobium halide glass‐ceramic electrolytes, LiX‐NbCl_5_ (X = F^−^, Cl^−^, Br^−^, I^−^). Specifically, the LiNbCl_6_ and LiNbCl_5_Br compositions exhibited high ionic conductivities of (3.46 ± 0.22) and (4.00 ± 0.20) mS cm^−1^ at 25°C, respectively, coupled with low activation energies of about 0.28 eV, indicative of facile lithium transport. Structural analysis confirmed predominantly amorphous phase(s) with embedded nanocrystalline domains, which likely contribute to their favorable transport properties. The slightly lower activation volume observed for LiNbCl_5_Br [(1.35 ± 0.07) cm^3^ mol^−1^] compared to that of LiNbCl_6_ [(1.40 ± 0.11) cm^3^ mol^−1^] correlates with its enhanced ion mobility. Leveraging their inherent oxidative (anodic) stability, when implemented as catholytes together with uncoated and LiNbO_3_‐coated Ni‐rich NCM cathode active materials, these electrolytes demonstrate high initial reversibility, with specific discharge capacities exceeding 200 mAh g^−1^ at C/10. In particular, cells employing the LiNbCl_5_Br exhibited superior cycling stability with 76% capacity retention after 100 cycles, highlighting the beneficial effect of partial Br^−^ substitution. This finding is supported by *ex situ* EIS measurements and distribution of relaxation times analysis on the evolution of interfacial resistance. Using differential electrochemical mass spectrometry, we also show to the best of our knowledge, for the first time, the release of gaseous chlorine at the end of charge (direct evidence of oxidative decomposition of Nb‐based halide SEs), in addition to oxygen and carbon dioxide. More importantly, the results indicate that the introduction of bromine helps reduce outgassing. While partial Br^−^ substitution does not strongly affect the bulk transport properties, it modifies the degradation pathways by suppressing chlorine evolution and improving interfacial stability. This lower gas evolution directly correlates with enhanced performance, highlighting the beneficial effect of partial Br^−^ substitution in this class of glass‐ceramic electrolytes.

Collectively, our findings underscore the potential of lithium niobium halides as catholytes for SSBs, with partial Br− substitution having a beneficial effect on cyclability (due to increasing electrochemical stability). However, further investigations into the nature of interfacial reactions and strategies for their mitigation are crucial to enhance the long‐term performance and practical viability of these materials, especially considering the possible release of corrosive and toxic gases during battery operation.

## Conflicts of Interest

The authors declare no conflicts of interest.

## Supporting information




**Supporting File**: smll74224‐sup‐0001‐SuppMat.docx.

## Data Availability

The original data for this paper is available at KITOpen. The data supporting this article have been included as part of the Supplementary Information. DOI for TEM data: https://doi.org/10.35097/0athhf23u67km8v1.
