# Supplementary material for: Improved Stability in LiX‐NbCl5 (X = Cl, Br) Glass‐Ceramic Electrolytes Through Anion Mixing for Solid‐State Batteries
Source: Small. 2026 Jun 17;22(45):e74224. doi: 10.1002/smll.74224 (PMC13472292; doi:10.1002/smll.74224)
Supplement: Supplementary file 1 [file SMLL-22-e74224-s001.docx]

**Supporting Information**

**Improved Stability in LiX-NbCl_5_ (X = Cl, Br) Glass-Ceramic Electrolytes Through Anion Mixing for Solid-State Batteries**

Jensheer Shamsudeen Seenath,^a^ Marvin Szabo,^b^ Philip Henkel,^a^ Rajib Sahu,^a,b^ Ramon Zimmermanns,^c,d^ Dhanush Yashwant Shanbhag,^a^ Christian Kübel,^b,d,e,f^ Stefanie Dehnen,^b^ Aleksandr Kondrakov,^a,g^ Torsten Brezesinski,^a^ and Florian Strauss^a^*

^a^Battery and Electrochemistry Laboratory (BELLA), Institute of Nanotechnology, Karlsruhe Institute of Technology (KIT), Kaiserstr. 12, 76131 Karlsruhe, Germany

^b^Institute of Nanotechnology, Karlsruhe Institute of Technology (KIT), Kaiserstr. 12, 76131 Karlsruhe, Germany

^c^Institute for Applied Materials-Energy Storage Systems, Karlsruhe Institute of Technology (KIT), Kaiserstr. 12, 76131 Karlsruhe, Germany

^d^Helmholtz Institute Ulm (HIU) Electrochemical Energy Storage, Helmholtzstr. 11, 89081 Ulm, Germany

^e^Karlsruhe Nano Micro Facility (KNMF), Karlsruhe Institute of Technology (KIT), Kaiserstr. 12, 76131 Karlsruhe, Germany

^f^Institute of Materials Science, Technical University Darmstadt (TUDa), Peter-Grünberg-Str. 2, 64287 Darmstadt, Germany

^g^BASF SE, Carl-Bosch-Str. 38, 67056 Ludwigshafen, Germany

*Corresponding author: [florian.strauss@kit.edu](mailto:florian.strauss@kit.edu)

**Experimental Section**

**General**

Both sample preparation and handling were carried out in a glovebox under Ar atmosphere (MBraun) with [O_2_] and [H_2_O] below 0.1 ppm. All precursors were used as received.

**Synthesis**

The precursor materials used in the preparation of LiNbCl_6_ and LiNbCl_5_Br were LiCl (99.9%; Sigma-Aldrich), LiBr (99.99%, anhydrous; Sigma-Aldrich), and NbCl_5_ (Thermo Fisher Scientific; 99.9%). LiCl was dried under vacuum (10^−3^ mbar) and at 300 °C overnight. A stochiometric precursor mixture was loaded into an 80 mL zirconia jar, along with 10 zirconia balls of 10 mm in diameter (~1.5 g total precursor mixture), and after premixing at 250 rpm for 1 h, the milling was continued at 450 rpm for 20 h. A similar procedure was also employed with the goal of preparing LiNbCl_5_F and LiNbCl_5_I. To this end, LiF (99.99%, anhydrous; Sigma-Aldrich) and LiI (99.999%, anhydrous; Sigma-Aldrich) were used as precursors. Annealing of LiNbCl_6_ was done under vacuum up to 200 °C for 2 h at a heating rate of 5 °C min^−1^.

**Characterization**

**Laboratory X-ray Diffraction (XRD).** Samples were sealed in borosilicate capillaries (0.68 mm inner diameter, 0.01 mm wall thickness; Hilgenberg) under Ar atmosphere, and XRD measurements were performed on a STOE Stadi-P diffractometer equipped with a DECTRIS MYTHEN 1K strip detector using a Mo anode to generate X-rays (*λ* = 0.70926 Å). Profile matching was done using the software FullProf Suite.

**Synchrotron X-ray Diffraction (SXRD), Total Scattering, and** **Pair Distribution Function (PDF) Analysis.** Synchrotron XRD and total scattering data was collected at room temperature at beamline P02.1, PETRA III at Deutsches Elektronen-Synchrotron (DESY) in Hamburg, Germany, with a wavelength of *λ* = 0.207371 Å. The exposure time for SXRD and total scattering was 60 and 600 s (10 shots of 60 s duration), respectively. The software PDFgetX3 was used to calculate *G*(r) from the raw total scattering data.^1^ The data was first corrected for sample container contribution and Compton scattering, and then, the normalized structure function *S*(Q) was obtained. Finally, *S*(Q) was Fourier transformed to yield *G*(r) with *Q*_max_ = 16 Å^−1^ and *r*_poly_ = 1.2 Å. Profile matching was done using the software FullProf Suite.

**Transmission Electron Microscopy (TEM).** Measurements were performed using a probe-corrected Thermo Fisher Titan Themis 300 operated at 300 kV. Elemental mapping was carried out in scanning TEM (STEM) mode using a Super-X energy-dispersive X-ray spectroscopy (EDS) detector, and high-resolution imaging was conducted in TEM mode. TEM specimens were prepared inside an Ar glovebox to prevent environmental degradation. Bulk powder was dispersed onto holey carbon-coated copper grids and transferred to the microscope utilizing a Gatan 648 vacuum transfer holder.

**Electrochemical Impedance Spectroscopy (EIS).** In order to measure the ionic conductivity, around 120 mg of solid electrolyte was loaded into a customized PEEK sleeve of diameter 10 mm. Stainless steel rods were then attached to both sides and the powder densified at a pressure of 3.5 t (437 MPa) for 2 min. EIS measurements were performed at 25 °C and under an external pressure of 250 MPa using a Biologic SP-200 potentiostat (0.1 Hz to 7 MHz frequency range, with a 20 mV voltage amplitude). The activation energy was calculated from Arrhenius plots by measuring the ionic conductivity from 15 to 65 °C, with at least 1 h of temperature equilibration at each individual measurement point.

**Direct Current (DC) Polarization.** The setup and assembling steps were similar to those described above for EIS. The DC polarization measurements were performed at 25 °C by applying voltages of 0.3, 0.4, and 0.5 V. The steady-state current was taken to determine the resistance and calculate the electronic conductivity.

**Linear Sweep Voltammetry (LSV).** To produce the solid electrolyte/cabon composite, solid electrolyte was mixed with Super C65 carbon black in a weight ratio of 7:3 at 140 rpm for 30 min, using a total amount of 1 g in an 80 mL zirconia jar, along with 10 zirconia balls of diameter 10 mm. For cell fabrication, 100 mg of Li_6_PS_5_Cl (LPSCl, NEI Corp.) was compacted at 62 MPa to form the separator layer. Then, around 20 mg of solid electrolyte/carbon composite was placed on one side (working electrode, WE), and the whole stack was pressed at 437 MPa for 2 min. Next, a 30 μm-thick In foil of diameter 9 mm (Zhejiang Haosheng New Material Co., Ltd.) was put onto the side facing the LPSCl, followed by 10 μm-thick Li foil of diameter 6 mm (China Energy Lithium Co., Ltd.) (counter electrode, CE). LSV measurements were performed at an external pressure of 81 MPa from OCV to 4.2 V vs. In/InLi (~4.82 V vs. Li^+^/Li) at a scan rate of 0.1 mV s^−1^ and at 25 °C.

**Pressure-Dependent Ionic Conductivity.** Pressure-dependent measurements were performed using the CompreDrive system (rhd instruments), with around 100 mg of solid electrolyte loaded into the 12-DP CompreCell (12 mm inner diameter). The pressure was applied uniaxially and maintained at a high accuracy (0.5%). A heating mantle (Eurotherm type ET3216) was employed to keep the temperature steady at 298 K, controlled by a Presto A40 thermostat (Julabo). No pressure was applied prior to the experiments, and the apparatus used had a lower pressure limit of 0.15 kN (1.3 MPa), with measurements initiated at 0.4 kN (3.5 MPa) till 650 MPa and with a step size of 25 MPa during the compression and vice versa for decompression. Acquired impedance spectra were analyzed using the software RelaxIS 3. Activation volumes were calculated by taking the logarithm of the ionic conductivity during the decompression step when the required linear behavior was observed, and the pellet was already fully densified.

**Electrode Preparation, Cell Assembly, and Battery Testing.** To assemble the SSB cells, customized PEEK sleeves (10 mm in diameter) with stainless steel dies were used. The cathode composite was first prepared by mixing either NCM851005 (85% Ni content, BASF SE) or LiNbO_3_-coated NCM851005 with solid electrolyte and Super C65 carbon black in a weight ratio of 69.3:29.7:1.0 at 140 rpm for 30 min, using a total amount of 1 g in an 80 mL zirconia jar, along with 10 zirconia balls of diameter 10 mm. The LiNbO_3_ protective coating on the NCM851005 secondary particle was prepared as reported in the literature.^2^

A bilayer separator was formed as follows. First, 70 mg of Li_6_PS_5_Cl (NEI Corp.) was pressed at 62 MPa, and another 30 mg of the glass-ceramic solid electrolyte was placed on one side of the pellet and cold pressed at 62 MPa. Then, around 12 mg of cathode composite was placed on the glass-ceramic solid electrolyte side, and the whole stack was compacted at 437 MPa for 2 min. Then, a 30 μm-thick In foil of diameter 9 mm (Zhejiang Haosheng Textile New Material Co., Ltd.) was put onto the side facing the Li_6_PS_5_Cl, followed by 10 μm-thick Li foil of diameter 6 mm (China Energy Lithium Co., Ltd.). SSB testing was done in the voltage range of 2.28-3.68 V vs. In/InLi (~2.9-4.3 V vs. Li^+^/Li) at 25 °C and under an external pressure of 81 MPa using a BioLogic potentiostat. Prior to the measurements, the cells were rested for 1 h at OCV. The C-rate was varied from 0.1 to 1C, with 1C = 190 mA g_NCM851005_^−1^.

**Differential Electrochemical Mass Spectrometry (DEMS).** For the DEMS measurements, SSB cells were prepared in a manner similar to those used for electrochemical testing, with the exception that only indium metal was used as anode. The cells were assembled in a 10 mm-diameter PEEK ring by sequentially pressing the individual components into a pellet before transferring them into the DEMS setup.^3^ Galvanostatic cycling was conducted at a rate of 0.05C and at 25 °C within a voltage window of 2.3-4.4 V vs. In/InLi, corresponding to approximately 2.9-5.0 V vs. Li^+^/Li, using a VSP-300 potentiostat (Biologic). To ensure temperature equilibrium and a stable background, all cells were held at OCV for 6 h prior to cycling. The helium carrier gas flow (2.5 mL min^−1^, 6.0 purity) was regulated by a mass flow controller (F-201CV-020-RAD-33-Z, Bronkhorst). Gas evolution was analyzed with a quadrupole mass spectrometer (OmniStar GSD 320 O, Pfeiffer Vacuum GmbH). After each measurement, a calibration gas was introduced to convert the measured ionic currents into molar ppm values.

**Figure S1.** Laboratory XRD patterns of (a) the different SEs after ball milling and (b) LiNbCl_6_ annealed at different temperatures. The positions of Bragg reflections for the precursors are indicated by vertical bars. The dashed lines in panel (b) indicate reflections related to LiCl.

**Figure S2.** (a) Nyquist plots from EIS measurements at 25 °C for the compositions used in the experiments to synthesize LiNbCl_5_I and LiNbCl_5_F. (b) Nyquist plots collected at 25 °C for LiNbCl_6_ and LiNbCl_5_Br and fitting obtained using the equivalent circuit model (inset).

**Figure S3.** SEM images of (a, b) LiNbCl_6_ and (c, d) LiNbCl_5_Br.

**Figure S4.** PDF analysis of the precursors and synthesized materials (LiNbCl_6_ and LiNbCl_5_Br). Data is shown up to *r* = 13 Å.

**Figure S5.** SAED patterns of the amorphous and nanocrystalline regions for (a, b) LiNbCl_6_ and (e, f) LiNbCl_5_Br. Determination of the layer spacing within the marked regions in (c, g) for (d) LiNbCl_6_ and (h) LiNbCl_5_Br.

**Figure S6**. Integrated EDS spectra showing the characteristic elemental peaks from Figures 1f and g.

**Figure S7.** TEM images at different magnifications of (a) LiNbCl_6_ and (b) LiNbCl_5_Br.

**Figure S8.** Nyquist plots obtained from the temperature-dependent EIS measurements for (a) LiNbCl_6_ and (b) LiNbCl_5_Br.

**Figure S9.** Pressure-dependent ionic conductivity during compression-decompression testing of (a) LiNbCl_6_ and (b) LiNbCl_5_Br.

**Figure S10.** Relative density as a function of pressure during compression and subsequent decompression for (a) LiNbCl_6_ and (b) LiNbCl_5_Br.

**Figure S11.** Cross-sectional SEM images of the solid electrolyte samples after pressure-dependent conductivity measurements for (a) LiNbCl_6_ and (b) LiNbCl_5_Br.

**Figure S12.** *I*-*V* polarization curves for (a) LiNbCl_6_ and (b) LiNbCl_5_Br.

**Figure S13.** Charge-discharge curves at C/20 and at 25 °C for SSB cells tested in the DEMS setup.

**Figure S14.** EIS data collected before and after cycling (100 cycles) from SSBs using LiNbO_3_-coated NCM851005 with (a) LiNbCl_6_ and (b) LiNbCl_5_Br.

**Table S1.** Summarized peak positions from the PDF data and assigned interatomic distances.

| Material | Peak position (Å) | Assignment |
| --- | --- | --- |
| NbCl_5_ | 2.3 | Nb-Cl |
|  | 3.89 | Nb-Nb |
| LiNbCl_6_ | 2.35 | Nb-Cl |
|  | 3.9 | Nb-Nb |
| LiNbCl_5_Br | 2.42 | Nb-Cl/Br |
|  | 3.92 | Nb-Nb |

**Table S2.** Summary of electrochemical performance metrics for SSBs with various halide-based SEs and CAMs. ICE: initial Coulomb efficiency. Missing values are indicated by as dash and if no C-rate was specified, the current density is given in units of mA cm^–2^ .

| **Cathode** | **Solid electrolyte** | **Voltage range**  **(V vs. Li^+^/Li)** | **ICE/Discharge capacity/C-rate** | **Discharge capacity/Cycle/ C-rate** | **Ref.** |
| --- | --- | --- | --- | --- | --- |
| LiNi_0.85_Co_0.1_Mn_0.05_O_2_ (NCM851005) | Li_2_Sc_2/3_Cl_4_ | 2.8–4.5 | - | 214/70/0.2C | 6 |
| LiNi_0.85_Co_0.1_Al_0.05_O_2_ (NCA) | Li_3_OCl | 3–4.5 | - | 100/250/0.5C | 7 |
| LiNi_0.8_Mn_0.1_Co_0.1_O_2_ (NCM811) | Li_2_ZrCl_6_ | 2.8–4.4 | 90.3/181/0.1C | 150/200/1C | 8 |
| NCM811 | Li_3_YCl_6_ | 3.1–4.3 | 87/181/0.1 mA cm^–2^ | 164.7/100/0.1 mA cm^–2^ | 9 |
| LiNi_0.5_Co_0.2_Mn_0.3_O_2_ (NCM523) | Li_0.388_Ta_0.238_La_0.475_Cl_3_ | 2.2–4.35 | 84.96/163/0.44C | 133.7/100/0.44C | 10 |
| LiNi_0.9_Co_0.05_Mn_0.05_O_2_ (NMC955) | Li_2.5_ZrCl_5_F_0.5_O_0.5_ | 2.5–4.35 | -/207.1/0.1C | 168/500/0.5C | 11 |
| LiCoO_2_ (LCO) | Li_3_InCl_6_ | 2.5–4.6 | 94.1/185.6/0.1C | 139.2/95/- | 12 |
| LiFePO_4_ | Li_2_(OH)_0.9_F_0.1_Cl | - | -/125/0.2C at 65 °C | 120/15/0.2C at 65 °C | 13 |
| LiNi_0.6_Co_0.2_Mn_0.2_O_2_ (NCM622) | Li_2_In_1/3_Sc_1/3_Cl_4_ | 2.8–4.6 | -/194/0.2C | 180/320/0.2C | 14 |
| LCO | Li_3_YCl_3_Br_3_ | 2.6–4.3 | 99.7/124/0.1C | -/55/0.1C | 15 |
| LiNi_0.88_Co_0.11_Mn_0.01_O_2_ (NCM88) | Li_2.5_Zr_0.5_In_0.5_Cl_6_ | 2.9–4.3 | 87.2/202/0.1C | -/100/0.5C | 16 |
| NCM622 | Li_2.7_Yb_0.7_Zr_0.3_Cl_6_ | 2.8–4.3 | -/170/0.2C | 136/150/0.2C | 17 |
| LiNi_0.83_Co_0.11_Mn_0.06_O_2_ (NCM83) | 1.6Li_2_O-TaCl_5_ | 2.5–4.3 | 93.5/189.5/0.1C | 128/300/1 C | 18 |
| LiNbO_3_ @ LiNi_0.85_Co_0.1_Mn_0.05_O_2_ (NCM851005) | LiNbCl_6_ | 2.9–4.3 | 94/206/0.1C | 96/100/0.5C | This work |
|  | LiNbCl_5_Br | 2.9–4.3 | 96/211/0.1C | 119/100/0.5C |  |

**References**

(1)	Juhás, P.; Davis, T.; Farrow, C. L.; Billinge, S. J. L. PDFgetX3: A Rapid and Highly Automatable Program for Processing Powder Diffraction Data into Total Scattering Pair Distribution Functions. *J. Appl. Crystallogr.* **2013**, *46*, 560–566.

(2)	Payandeh, S.; Strauss, F.; Mazilkin, A.; Kondrakov, A.; Brezesinski, T. Tailoring the LiNbO₃ Coating of Ni-Rich Cathode Materials for Stable and High-Performance All-Solid-State Batteries. *Nano Res. Energy* **2022**, *1*, 9120016.

(3)	Bartsch, T.; Strauss, F.; Hatsukade, T.; Schiele, A.; Kim, A.; Hartmann, P.; Janek, J.; Brezesinski, T. Gas Evolution in All-Solid-State Battery Cells. *ACS Energy Lett.* **2018**, *3*, 2539–2543.

(4)	Zhao, Y.; Tenhaeff, W. E. Thermally and Oxidatively Stable Polymer Electrolyte for Lithium Batteries Enabled by Phthalate Plasticization. *ACS Appl. Polym. Mater.* **2020**, *2*, 80–90.

(5)	Bron, P.; Roling, B.; Dehnen, S. Impedance Characterization Reveals Mixed Conducting Interphases between Sulfidic Superionic Conductors and Lithium Metal Electrodes. *J. Power Sources* **2017**, *352*, 127–134.

(6)	Zhou, L.; Kwok, C. Y.; Shyamsunder, A.; Zhang, Q.; Wu, X.; Nazar, L. F. A New Halospinel Superionic Conductor for High-Voltage All-Solid-State Lithium Batteries. *Energy Environ. Sci.* **2020**, *13*, 2056–2063.

(7)	Yan, C.; Xu, R.; Qin, J.-L.; Yuan, H.; Xiao, Y.; Xu, L.; Huang, J.-Q. 4.5 V High-Voltage Rechargeable Batteries Enabled by the Reduction of Polarization on the Lithium Metal Anode. *Angew. Chem.* **2019**, *131*, 15379–15382.

(8)	Wang, K.; Ren, Q.; Gu, Z.; Duan, C.; Wang, J.; Zhu, F.; Fu, Y.; Hao, J.; Zhu, J.; He, L.; et al. A Cost-Effective and Humidity-Tolerant Chloride Solid Electrolyte for Lithium Batteries. *Nat. Commun.* **2021**, *12*, 4410.

(9)	Ji, W.; Zheng, D.; Zhang, X.; Ding, T.; Qu, D. A Kinetically Stable Anode Interface for Li₃YCl₆-Based All-Solid-State Lithium Batteries. *J. Mater. Chem. A* **2021**, *9*, 15012–15018.

(10)	Yin, Y.-C.; Yang, J.-T.; Luo, J.-D.; Lu, G.-X.; Huang, Z.; Wang, J.-P.; Li, P.; Li, F.; Wu, Y.-C.; Tian, T.; et al. A LaCl₃-Based Lithium Superionic Conductor Compatible with Lithium Metal. *Nature* **2023**, *616*, 77–83.

(11)	Shen, L.; Li, J.-L.; Kong, W.-J.; Bi, C.-X.; Xu, P.; Huang, X.-Y.; Huang, W.-Z.; Fu, F.; Le, Y.-C.; Zhao, C.-Z.; et al. Anion-Engineering Toward High-Voltage-Stable Halide Superionic Conductors for All-Solid-State Lithium Batteries. *Adv. Funct. Mater.* **2024**, *34*, 2408571.

(12)	Wang, Z.; Tan, J.; Jia, Z.; Cui, J.; Wang, X.; Shu, C.; Gao, X.; Wu, Y.; Tang, W. Deciphering Chemical/Electrochemical Compatibility of Li₃InCl₆ in 5.2 V High-Voltage LiCoO₂ All-Solid-State Batteries. *ACS Energy Lett.* **2024**, *9*, 4485–4492.

(13)	Li, Y.; Zhou, W.; Xin, S.; Li, S.; Zhu, J.; Lü, X.; Cui, Z.; Jia, Q.; Zhou, J.; Zhao, Y.; et al. Fluorine-Doped Antiperovskite Electrolyte for All-Solid-State Lithium-Ion Batteries. *Angew. Chem. Int. Ed.* **2016**, *55*, 9965–9968.

(14)	Zhou, L.; Zuo, T.-T.; Kwok, C. Y.; Kim, S. Y.; Assoud, A.; Zhang, Q.; Janek, J.; Nazar, L. F. High Areal Capacity, Long Cycle Life 4 V Ceramic All-Solid-State Li-Ion Batteries Enabled by Chloride Solid Electrolytes. *Nat. Energy* **2022**, *7*, 83–93.

(15)	Liu, Z.; Ma, S.; Liu, J.; Xiong, S.; Ma, Y.; Chen, H. High Ionic Conductivity Achieved in Li₃Y(Br₃Cl₃) Mixed Halide Solid Electrolyte via Promoted Diffusion Pathways and Enhanced Grain Boundary. *ACS Energy Lett.* **2021**, *6*, 298–304.

(16)	Kwak, H.; Han, D.; Son, J. P.; Kim, J. S.; Park, J.; Nam, K.-W.; Kim, H.; Jung, Y. S. Li⁺ Conduction in Aliovalent-Substituted Monoclinic Li₂ZrCl₆ for All-Solid-State Batteries: Li₂₊ₓZr₁₋ₓMₓCl₆ (M = In, Sc). *Chem. Eng. J.* **2022**, *437*, 135413.

(17)	Kim, S. Y.; Kaup, K.; Park, K.-H.; Assoud, A.; Zhou, L.; Liu, J.; Wu, X.; Nazar, L. F. Lithium Ytterbium-Based Halide Solid Electrolytes for High Voltage All-Solid-State Batteries. *ACS Mater. Lett.* **2021**, *3*, 930–938.

(18)	Zhang, S.; Zhao, F.; Chen, J.; Fu, J.; Luo, J.; Alahakoon, S. H.; Chang, L. Y.; Feng, R.; Shakouri, M.; Liang, J.; et al. A Family of Oxychloride Amorphous Solid Electrolytes for Long-Cycling All-Solid-State Lithium Batteries. *Nat. Commun.* **2023**, *14*, 3780.
